# Root osmotic sensing from local perception to systemic responses

**DOI:** 10.1007/s44154-022-00054-1

**Published:** 2022-09-05

**Authors:** Lucille Gorgues, Xuelian Li, Christophe Maurel, Alexandre Martinière, Philippe Nacry

**Affiliations:** IPSiM, CNRS, INRAE, Institut Agro, Univ Montpellier, 34060 Montpellier, France

**Keywords:** Drought, Water deficit perception, Local signaling, Long distance signaling, Local water deficit, Adaptive development

## Abstract

Plants face a constantly changing environment, requiring fine tuning of their growth and development. Plants have therefore developed numerous mechanisms to cope with environmental stress conditions. One striking example is root response to water deficit. Upon drought (which causes osmotic stress to cells), plants can among other responses alter locally their root system architecture (hydropatterning) or orientate their root growth to optimize water uptake (hydrotropism). They can also modify their hydraulic properties, metabolism and development coordinately at the whole root and plant levels. Upstream of these developmental and physiological changes, plant roots must perceive and transduce signals for water availability. Here, we review current knowledge on plant osmotic perception and discuss how long distance signaling can play a role in signal integration, leading to the great phenotypic plasticity of roots and plant development.

## Introduction

Like some prokaryotic and most fungal cells, plant cells are wall-encapsulated. Although it provides important advantages such as a robust exoskeleton and a protection of cells from adverse abiotic or biotic factors, the wall creates a direct restriction for cellular expansion. This important constraint can be overcome by intracellular turgor that in response to cell wall loosening allows growth. The cell turgor, that can be up to 10 bars in certain cell types, is actually built from the osmotic gradient between cell interior and the external media. This gradient triggers a flux of water into the cell that leads to a compensatory hydrostatic pressure, called turgor. Any change in the osmotic gradient by for instance an increase/decrease in water potential in the external media or changes in the internal solute concentration, leads to a direct change in turgor that can modify cell volume and tissue rigidity. To allow localized expansion growth or prevent wilting, cells have therefore to maintain a constant dialog between cell wall mechanical properties, solute concentration and turgor.

Whereas plant aerial parts are protected from air drying by specialized waxes deposited on their surface, the root system is in direct contact with its surrounding environment and therefore has to cope with dramatic changes in water potential. Soil is a complex porous medium with marked differences in its composition and structure e.g. air pockets and hydrated soil particles. Thus, root systems are continuously facing contrasted water availabilities during soil exploration. In addition to a local perception of water availability, followed by signal transduction and responses at cellular level, long-distance signals are produced. These so-called systemic signals coordinate responses at the multicellular scale, to modulate integrative traits, such as root system architecture (RSA).

In this review, we will focus on recent advances in the field of plant osmotic perception and early signaling in roots. We will also underline how systemic signaling can integrate local signals to modulate RSA.

## Local perception and early signaling

**Fig. 1:**
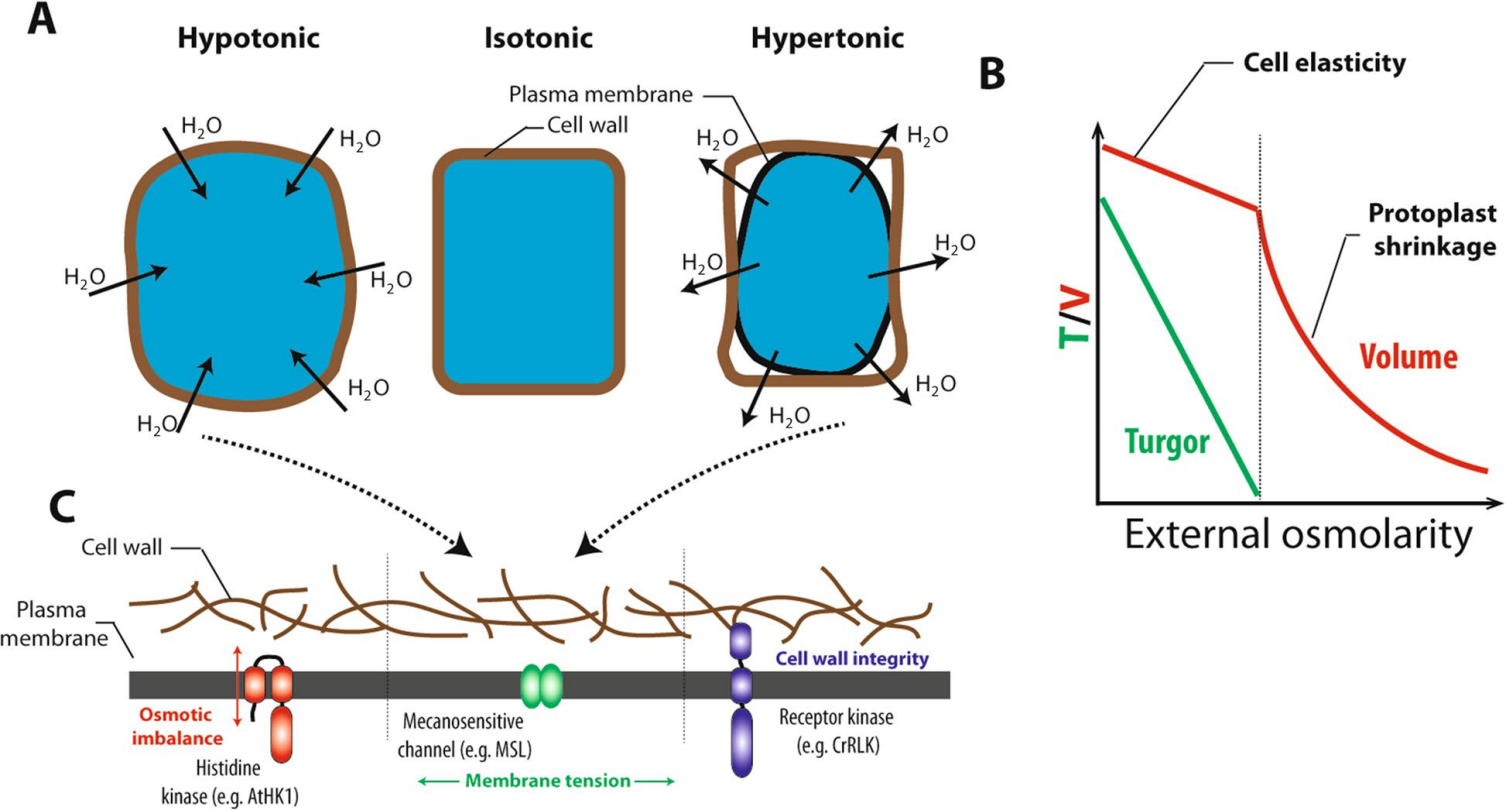
Nature of the osmotic signal and suspected sensing mechanisms. **A** Drawing of the relation between osmotic, water fluxes and cellular volume regulation. A reduced (hypotonic) or increased (hypertonic) external osmolarity results in an influx or efflux of water, respectively. Depending on cell wall elasticity, these fluxes lead to changes in cellular volume. **B** Relative variations of cell turgor and volume in response to an increase of external osmolarity. In the absence of cellular osmoregulation, the turgor tends to decrease linearly with increasing osmolarity. In contrast, the cellular volume is expected to decrease in a two-phase mode, a quasi-linear mode as long as turgor is maintained in the cell, followed by a hyperbolic decay when turgor is absent. **C** Based on the literature 3 classes of perception mechanism can sense the osmotic signal. Osmotic signal may be perceived at the membrane from either a local osmotic imbalance (e.g. AtHK1) or a change in membrane tension (e.g. MSL) or from a perturbation of cell wall integrity (e.g. CrRLK)

Although being searched for many years, the exact nature of the signals that allow plants to perceive changes in water availability is not clearly defined yet. At the cellular level, mechanics e.g. cell wall/membrane tension, but also cell or subcellular volumes are directly impacted by changes in external osmolarity (Fig. 1A and B). Historically, comparison between different kingdoms has permitted the identification of some molecular players. Since osmotic perception is mandatory all along the plant life cycle, it is likely that many molecular mechanisms are actually superimposed in cells (Fig. 1C). New approaches, especially genetic screens, have recently allowed a striking expansion of knowledge on plant osmotic perception, which is summarized and updated in the present paragraph.

### Molecular mechanisms of perception

#### Histidine kinases (AtHK1)

Historically, the first osmosensing pathway has been uncovered in unicellular organisms. In *Saccharomyces cerevisiae*, perception is made by a two-component histidine kinase system, where recognition of the stimulus leads to kinase activation and phosphorylation of a histidine residue (Brewster and Gustin 1994). The Synthetic Lethal of N-end rule 1 (SLN1) changes its phosphorylation activity in response to osmotic signal and activates via several phosphorelays the High Osmolarity Gycerol (HOG1) MAP kinase (Ota and Varshavsky 1993; Maeda et al. 1995). In turn, HOG1 controls the expression of osmotic responsive genes including those involved in the biosynthesis of compatible osmolytes like glycerol.

**Fig. 2:**
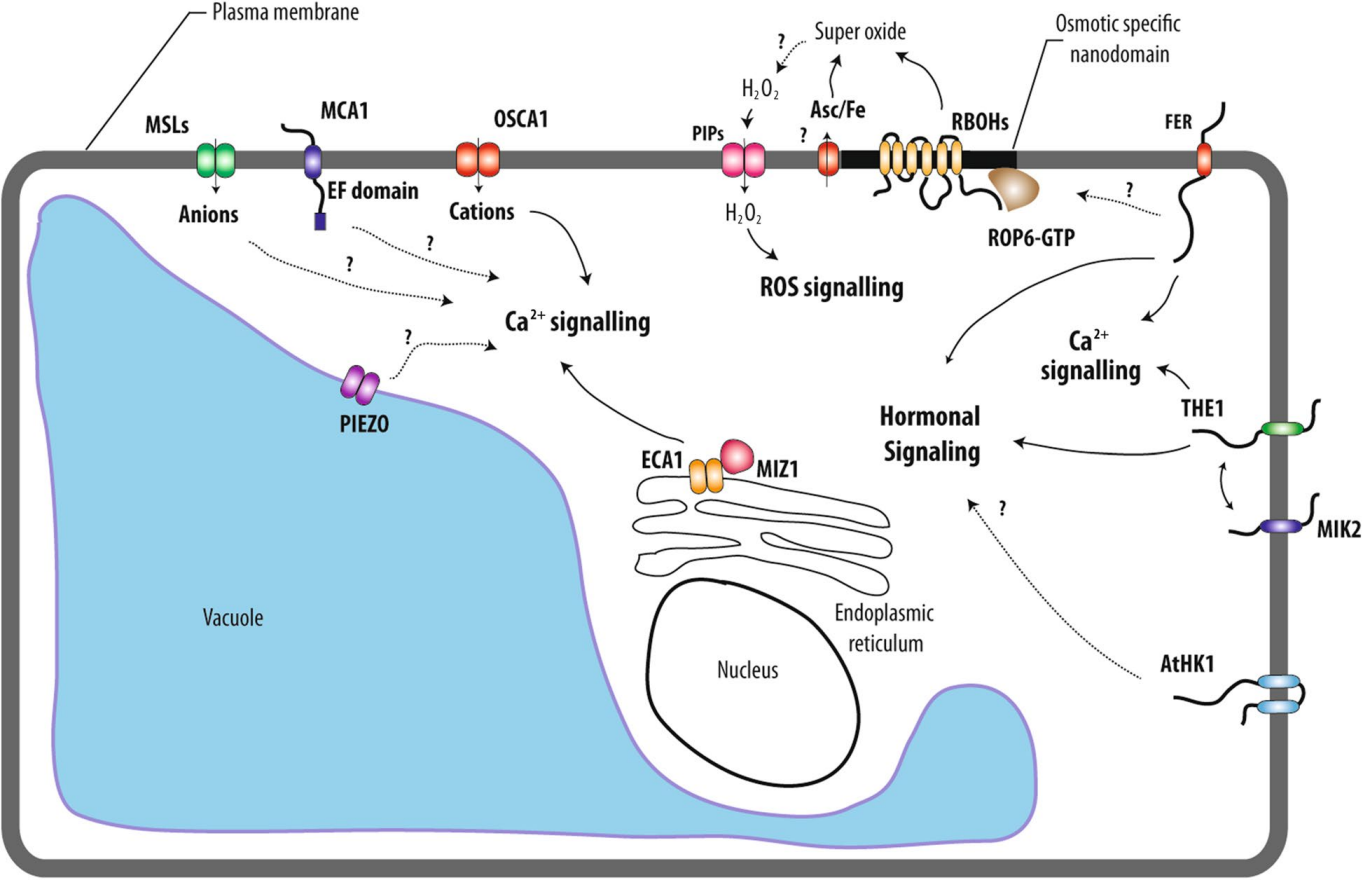
Summary of currently known osmotic perception mechanisms in plants. Changes in membrane tension induced by osmolarity imbalance can be perceived by membrane mechanosensors such as OSCA1, MSLs, MCA1, PIEZO, ECA1/MIZ1. By transporting cations or anions, these sensors initiate cell calcium signaling by as yet unknown mechanisms. Receptor-like kinases belonging to the CrRLK family (e.g. FER, THE) perceive the cell wall status and their activation eventually leads to cell wall reinforcement. Whereas their exact role as osmotic sensors has yet to be established, these receptors definitely fine tune signaling of hormones such as ABA, auxin and jasmonate that are known to regulate plant development and physiological acclimation to osmotic stress. At the cell membrane, a partial integration of signals can be observed. For instance, LRR kinase MIK2, a receptor for phytocytokines that controls plant immunity, genetically interacts with THE, pointing to a link between osmotic and pathogen signaling. By similarity to the yeast system, the AtHK1 two component histidine kinase may also participate in osmotic signaling by modulating ABA signaling. In addition to hormones and calcium signaling, Reactive Oxygen Species (ROS) are also participating in early cell responses to osmotic stimuli. Cellular accumulation of ROS is dependent on NADPH oxidases (RBOHD and F) and iron reduction processes. Upon cell stimulation, ROP6 forms nanodomains together with the superoxide producing enzyme RBOHD/F. As a consequence, superoxide can be dismutated to hydrogen peroxide (H_2_O_2_) by apoplastic SOD (Superoxide dismutase). H_2_O_2_ transport through the cell membrane is in turn facilitated by aquaporins

Plants also show a conserved gene family of histidine kinases (HK) with members involved in hormonal signaling such as ethylene (Gamble et al. 2002) or cytokinins. The Arabidopsis genome contains 11 genes coding for Histidine kinase like protein (Hutchison and Kieber 2002). In addition to its role in hormone signaling, Arabidopsis AtHK1 was shown to act as an osmosensor by complementing yeast *sln1* mutation (Urao et al. 1999) (Figs. 1C and 2). Localized to the plasma membrane, AtHK1 is a positive regulator of salt stress, drought and ABA signaling (Tran et al. 2007; Wohlbach et al. 2008). For instance, overexpression AtHK1 in plants increases the drought tolerance and results in the induction of genes involved in drought signaling including proline and sucrose biosynthesis (Tran et al. 2007). However, the role of AtHK1 as a main plant osmosensor has been questioned. Indeed, in several loss-of-function alleles of *athk1,* no hyperosmotic signaling-related phenotypes such as ABA, or osmolyte accumulation were found **(**Kumar et al. 2013).

#### Mechanosensitive channels (MSL, OSCA1, Piezo, …)

Due to its molecular anchors with the wall and the absence of folding, the plant plasma membrane of turgid cells is potentially under tension, especially during cell expansion and osmotic volume adjustment. Changes in membrane tension due to an osmotic challenge can therefore be perceived by cells through membrane mechanosensitive channels (Fig. 1C). The first mechanosensors involved in osmoregulation were molecularly identified from *Escherichia coli* spheroplasts (Martinac et al. 1987; Levina 1999). Several studies have solved the crystal structure of these low-conductance mechanosensors. They highlighted 3 common transmembrane helices that would serve as sensors of the membrane tension (Bass et al. 2002; Steinbacher et al. 2007; Lai et al. 2013). In addition, two recent cryomicroscopy studies have shown that *E. coli* MscS channels (MscS) association with lipids is an important determinant for the stabilization of the closed conformation and therefore plays a key role in channel sensitivity to tension (Flegler et al. 2021; Zhang et al. 2021b). The gating of *E. coli* MscS channels is conditioned by a pocket containing lipids. The anisotropic forces of the membrane created by tension could free this pocket of lipids or/and change the number of lipid acyl chains in it, causing a structural rearrangement and leading to pore opening (Pliotas et al. 2015; Flegler et al. 2021).

In plants, members of the MscS-like (MSL) gene family represent the closest homologs of bacterial MscS (Sukharev et al. 1994). MSLs are non-selective anion channels whose activity is regulated by voltage or mechanical stimulation of the membrane (Haswell 2007). Ten MSL have been described in Arabidopsis (Haswell 2007). In a landmark study, MSL9 and 10 were shown to control mechanosensitive activity of anion channels in the plasma membrane of root protoplasts (Haswell et al. 2008) (Fig. 2). Because neither the double *msl9/10* nor the quintuple *msl4/5/6/9/10* mutant showed alteration of their response under a variety of stimuli, e.g. osmotic, salt, mechanical or dehydration, the exact integrated function of these channels has remained enigmatic (Haswell et al. 2008). Whereas, MSL10 was demonstrated to be a true mechanosensitive channel after heterologous expression (Maksaev and Haswell 2012), its function has been recently uncovered using an elegant experimental strategy. By playing both with cell wall inhibitor that loose the cell wall and osmoticum to modulate cell turgor, Maksaev and co-authors show that MSL10 potentiate cell responses to swelling (Basu et al. 2020). Hypoosmotic shock induced several responses including cytoplasmic calcium increases, ROS accumulation and transcriptional regulation that are under the control of MSL10 (Basu et al. 2020). In addition, this channel determines the induction of programmed cell death (PCD) in response to a hypo-osmotic challenge. This response is dependent on the phosphorylation of MSL10 N-terminus (Veley et al. 2014; Basu et al. 2020; Basu and Haswell 2020). In a recent preprint, MSL10 was found to localize and function at ER-plasma membrane contact sites (EPCSs) (Codjoe et al. 2022). In plants, EPCSs regulate lipid homeostasis between compartment and cell integrity (Schapire et al. 2008; Ruiz-Lopez et al. 2021). Those results suggest an interesting connection between mechanosensitive channel and regulation of cell volumes. MSLs are also important for regulation of organelle volume within cells. MSL2 and MSL3 that localize to plastid membranes, modulate their division, shape and size (Lee et al. 2019; Wilson et al. 2016). Because *msl2/3* plants develop a mass callus tissue at their shoot apical meristem, MSLs create a link between plastid osmoregulation and cell differentiation. MSLs were also associated with specific cell types like pollen tubes (Hamilton et al. 2015). MSL8 was shown to prevent cell bursting during pollen rehydration processes and membrane integrity during tip growth; highlighting again the role of MSLs during hypoosmotic choc. However, a recent preprint suggests that MSL8 effect on pollen tube hydration is not due to a simple tension-gated osmoregulator. MSL8 would rather regulate ions fluxes that are needed for cell wall modification during pollen hydratation (Miller et al. 2022).

Other types of mechanosensitive channels have been identified in Arabidopsis. Mid1-Complementing Activity (MCA1) was cloned by complementing the lethal mutation of *mid1* yeast lacking the putative Ca^2+^ permeable stretch-activated channel component (Nakagawa et al. 2007 PNAS). MCA1 and its Arabidopsis paralog, MCA2*,* exhibited a Ca^2+^ permeable mechanosensitive channel activity in Xenopus (Furuichi et al. 2012 PSB). Interestingly, MCAs consist of single pass transmembrane proteins (Kamano et al. 2015). Their first 200 amino acids contain both the TM and the EF domains that are sufficient for channel activity under membrane stretching (Yoshimura et al. 2021). MCA loss of function mutants show root growth defects in media with high concentration of agar and a hypersensitivity to cold stress (Nakagawa et al. 2007; Mori et al. 2018) (Fig. 2). However, *mca1 mca2* showed no alteration in calcium influx after osmotic stimulation, questioning the role of the two channels in the osmosensing pathway (Stephan et al. 2016).

Members of the PIEZO gene family, from the Greek “pι’esi” which means pressure, are plasma membrane localized cation channels involved in mechanosensory processes and necessary for light touch perception, compressive force proprioception, among other processes. Their discovery was recognized by the 2021’s Nobel prize award. Arabidopsis genome encode for unique PIEZO homolog. at the AtPIEZO channel was first identified as a regulator of virus spreading and of root cap mechanotransduction (Mousavi et al. 2021; Zhang et al. 2019b). However, AtPIEZO1 seems to also act in tip growing cells. The two homologues encoded by the moss *Physcomitrella patens* genome are localized in the tonoplast and control cell growth and cytoplasmic calcium oscillation (Radin et al. 2021). Analysis of loss and gain of function mutants showed that moss PIEZOs act on the vacuole membrane through tubulation, internalization or fission (Radin et al. 2021). AtPIEZO is also localized in the tonoplast and acts on vacuole morphology of pollen tube; showing that PIEZO function on tip growing cells is conserved among land plants (Radin et al. 2021). These observations shade light onto the role of the plant vacuole in mechanoperception and maybe osmosensing (Fig. 2).

In addition to searching in the plant genome for homologs of prokaryotic or eukaryotic mechanosensors, direct genetic screens have been realized to identify plant osmosensors. Calcium influx in the cytoplasm is probably one of the fastest cell responses to osmotic stimulation. Following a calcium imaging screen, the gene REDUCED HYPEROSMOLALITY-INDUCED [CA^2+^] INCREASE 1/ (OSCA1.1) was therefore identified from an EMS mutagenized population. OSCA1.1 encodes for a hyperosmolality-gated calcium-permeable channel, which is responsible for calcium signaling, but also regulation of root growth, stomata closure and transpiration upon osmotic stimulation (Yuan et al. 2014) (Fig. 2). Interestingly, OSCA1 is not required for ABA- or H_2_O_2_-induced calcium cellular influxes suggesting a strong specificity of the channel for osmotic signaling. Arabidopsis Calcium-permeable Stress-gated cation Channel 1 (AtCSC1/OSAC1.2), a close homolog of OSCA1, is also responsible for osmotic-dependent calcium influx when expressed in Chinese hamster ovary (CHO) cells (Hou et al. 2014).

Atomic structure and cryo-microscopy analyses have shown that OSCA1.2 is composed of 11 transmembrane helices forming homodimers. This channel has a cytosolic domain with RNA recognition motifs and 2 alpha-helices anchoring the protein in membrane lipids (Jojoa-Cruz et al. 2018; Liu et al. 2018). The two additive helices are cytosolic and might serve as a lateral sensor for tension within the inner plasma membrane leaflet (Maity et al. 2019). Except for the OSCA4.1 isoform belonging to clade 4, the mechanosensitive channel activity seems to be conserved among the OSCA gene family when expressed in HEK cells or in proteoliposomes (Murthy et al. 2018). However, the isoforms present distinct ion conductances and sensitivities to pressure (Murthy et al. 2018). Knowledge on the role of OSCAs in planta has recently been expanding. For instance, OSCA1.3 behaves as a Ca^2+^ permeable channel in yeast and contributes, with its close homologue OSCA1.7, to pattern-triggered immunity (PTI)-dependent stomatal closure (Thor et al. 2020). Thus, OSCA function in plants is not strictly restricted to mechano or osmoperception.

#### Cell wall sensing (CrRLKs, WAKs, …)

Since the cell wall is way stiffer than the membrane, most of the tensile stress induced by turgor is borne by the wall. Recently, knowledge of biological processes that perceive the cell wall status has rapidly expanded. A number of transmembrane receptor like kinases (RLKs) such as *Catharanthus roseus* RLK1-Like kinases (CrRLK), Wall Associated Kinases (WAK), Lectin Receptor-Kinase (LRK), Proline-rich Extensin-like Receptor Kinases (PERKs), and formins have been shown to interact with the cell wall (Wolf et al. 2012). Some of these are known to act as sensors of the cell wall status. Historically, the *THESEUS* (THE) CrRLK was isolated from a genetic screen for suppression of the short hypocotyl and ectopic lignin phenotype of the cell wall synthase mutant *cesa6*^*prc1–1*^ (Hématy et al. 2007). Indeed, the retarded growth induced by inhibition of cellulose synthesis is not the direct result of structural changes in the cell wall linked to reduce cellulose content. It rather involves active inhibition of growth through receptor kinases like THE (Hématy et al. 2007). In addition to its role as a wall integrity sensor, THE acts on lateral root initiation through perception of its ligand, RALF34 (Gonneau et al. 2018). THE1 signaling is also needed to control ABA accumulation after osmotic stress (Bacete et al. 2022) (Fig. 2). These results illustrate the intricacy between wall integrity sensors, the maintenance of cell mechanics and ABA signaling as a typical osmotic signaling marker.

Interestingly, THE also controls some salt-induced related phenotypes, like root skewing. This response also needs MALE DISCOVERER 1-INTERACTING RECEPTOR-LIKE KINASE 2 (MIK2). This LRR kinase was identified from a reverse genetic screen for impaired ectopic lignin deposition under treatment with isoxaben, an inhibitor of cellulose deposition (Van der Does et al. 2017). Allelic variation in *MIK2* has been associated with changes in rosette dry weight in response to mild salt (Julkowska et al. 2016) (Fig. 2). MIK2 binds to SERINE RICH ENDOGENOUS PEPTIDE12 (SCOOP12), a phytocytokine that is secreted and regulates immunity in plants (Hou et al. 2021; Rhodes et al. 2021). These observations suggest an interaction between cell wall sensing pathways, including response to osmotic stimulation, and plant immune responses.

FERONIA (FER) is another CrRLK that participates in cell wall sensing. Loss-of-function studies have revealed that FER is involved in many processes such as cell elongation, root hair development, responses to hormones, nutrition and plant defense (Li et al. 2016). In addition, loss-of-function studies of FER have shown defects in responses to mechanical stimuli e.g. inability to penetrate hard layers of agar (Shih et al. 2014). FER has also a tight link to ABA signaling and loss of function plants show a resistant phenotype to osmotic stress and a hyper sensitivity to salt stress, although salt stress also results in osmotic stress (Chen et al. 2016 PNAS) (Fig. 2). Feng and co-authors also highlighted the specific role of FER in response to salt stress (Feng et al. 2018). FER induces relatively late calcium influx in the cell that is needed to sustain cell wall reinforcement. It was proposed that FER would sense the impact of sodium ions on pectin filament organization, rather than directly sensing turgor or plasmolysis (Feng et al. 2018).

### Molecular mechanisms of early signaling

#### Calcium

Calcium is a secondary messenger that allows the transmission of many biotic and abiotic stimuli and converts them into cellular signals. Intracellular calcium homeostasis is modulated by channels, pumps and transporters. The calcium signal is transmitted by calcium-binding proteins that subsequently feed into signaling cascades (Dodd et al. 2010; Kudla et al. 2010). Fluorescent Ca^2+^ probes have been actively used to identify quantitative characteristics like amplitude or frequency of calcium responses; revealing the concept of calcium signatures for specific signals (Monshausen 2012). According to this, a pure osmotic stimulus induces intracellular calcium signals that are different from those induced by stimuli like cold or salt, that are only partially composed of an osmotic component (Tracy et al. 2008; Huang et al. 2017a). In addition, Ca^2+^ varies also in space within the cell, with the nucleus and cytoplasm responding differently to osmotic stimulation probably through independent molecular mechanisms (Huang et al. 2017a; Luo et al. 2020). Whereas OSCA1 appears as a crucial actor of osmotically driven calcium influx, the exact role of other mechanosensitive channels like MSLs or MCAs remains debated (Yuan et al. 2014; Stephan et al. 2016). Interestingly, intracellular compartments also play critical roles in controlling calcium signaling in response to osmotic signals. The K^+^ exchange antiporters (KEAs), which perform plastid localized potassium antiport, are needed to maintain plastid ion homeostasis. In corresponding loss of function plants, plastids were swollen and had impaired calcium influx after osmotic stimulation (Stephan et al. 2016). Nevertheless, the link between plastid ion homeostasis and calcium signaling remains to be explored. In addition, calcium signal decoding processes in response to osmotic signals remain largely uncharacterized.

At the root tissue level, imaging approaches have identified calcium movements according to water potential gradient (Shkolnik et al. 2018). The calcium flux propagates along the phloem until it reaches the root elongation zone to control hydrotropic responses, i.e. directed root growth towards higher soil water potentials (Shkolnik et al. 2018). Both the osmotic stress-induced calcium increase and hydrotropic response need functional ECA1, an endoplasmic reticulum Ca^2+^ pump, and MIZU-KUSSEI1 (MIZ1) (Fig. 2). The latter gene was originally identified from a forward genetic screen based on hydrotropic response (Kobayashi et al. 2007). These data suggest that calcium participates as a secondary messenger at the cellular level but also at distances allowing hydrotropism.

#### ROS

In addition to calcium, reactive oxygen species (ROS) accumulate minutes after cell stimulation (Leshem and Levine 2007; Martinière et al. 2019). Superoxide, hydrogen peroxide, but also other ROS are generated from both cellular metabolism and through specific generator systems. Indeed, ROS not only represent stress-induced damaging components, but also serve as genuine cell secondary messengers (Devireddy et al. 2021; Fichman and Mittler 2020). Upon an osmotic signal, ROS was linked to root hydrotropic responses. Indeed, inhibition of peroxidases and Respiratory Burst Oxidase Homolog family (RBOHs), which are classical ROS consumer and producer respectively, induce altered root curvature when plants are grown on plate containing a water potential gradient (Krieger et al. 2016; Jiménez-Nopala et al. 2018). In fact, it was found that ROS accumulation in the elongation zone participate to root gravitropic response, maybe through regulation of autophagy and amyloplast degradation (Nakayama et al. 2012; Krieger et al. 2016). This agravitropic phenotype leads to a stronger hydrotropic response. Then, ROS function in tuning root tropic responses by promoting gravitropism and therefore negatively regulating hydrotropism response. Strikingly, the RBOHD and F can only partially account for the osmotically-induced ROS production. Indeed, upon signal, the cytoplasmic reducing power is transferred to the apoplasm, leading to ascorbate accumulation. Ascorbate is further used to reduce iron that reacts with di-oxygen to generate superoxide (Martinière et al. 2019). In cells, the ROS generated by RBOHs or the ascorbate/iron pair end up with different responses (Fig. 2). Whereas the two ROS generating pathways are needed for membrane internalization, RBOH-dependent ROS induces the internalization of specific cargo proteins such as the PIP2;1 aquaporin (Martinière et al. 2019). Interestingly, certain ROS species have the particularity of being able to travel through cell compartments. For instance, membrane transport of hydrogen peroxide (H_2_O_2_) is facilitated by certain aquaporins localized at the PM (PIP1–4) (Tian et al. 2016), PIP2;1 (Rodrigues et al. 2017) or the tonoplast e.g. TIP1;1 and TIP1;2 (Bienert et al. 2007) (Fig. 2). Therefore, H_2_O_2_ might itself regulate its own membrane permeability by acting on aquaporin cycling.

Recently, Rho of Plant6 (ROP6) was found to be necessary and sufficient to induce osmotically-induced ROS production (Smokvarska et al. 2020). Rho-GTPases (RAC/ROP) are involved in cell responses to various stimuli such as auxin, ABA orchitin elicitation (Feiguelman et al. 2018). ROPs are molecules that switch from an inactive to an active form by binding to GDP or GTP, respectively (Feiguelman et al. 2018). Models linking ROPs and NADPH oxidase have been described for root hair or pollen growth and immune response (Duan et al. 2010; Boisson-dernier et al. 2013). After osmotic stimulation, ROP6 is activated and recruited in PM domains of nanometric size, together with RBOHD and F (Fig. 2). Interestingly, the co-recruitment of RBOHs within nanodomains seems to be specific for the osmotic signal. To convey auxin signaling, ROP6 has also to form nanodomains, which however are exempt of RBOHs. As a result, no ROS accumulates in cells under auxin treatment (Platre et al. 2019; Smokvarska et al. 2020). These observations suggest that the ROP6 activation mechanism can itself control the specificity of the downstream response.

Osmotically-induced ROS are associated with numerous downstream cellular and physiological responses such as a decrease in root hydraulic conductivity or accumulation of compatible osmolytes like proline (Boursiac et al. 2008; Ben Rejeb et al. 2015). Detoxification mechanisms through specific enzymes like superoxide dismutases (SOD), ascorbate peroxidases (APX), catalases (CAT), glutathione peroxidases (GPX) and peroxiredoxins (PRX) (Mittler et al. 2004) are needed. For instance, AtGPX3/GPXL3 was linked to drought stress and ABA signaling. *Atgpx3/gpxl3* plants showed enhanced water losses under drought stress (Miao et al. 2006). In vitro phosphorylation assays showed that oxidized AtGPX3/GPXL3 acts on phosphatase activity of ABA INSENSITIVE2 (ABI2) (Miao et al. 2006). Consistently, AtGPX3/GPXL3 links ABA signaling, ROS and osmotic signaling. Because the AtGPX3/GPXL3 catalytic domain is facing the lumen of the secretory pathway, it remains muddled how AtGPX3/GPXL3 oxidation is mechanistically associated to ABI2 (Attacha et al. 2017).

## Systemic signaling and developmental responses

To cope with their complex and fluctuating environment, plants have to constantly sense the surrounding fluctuations and integrate all this local information into coordinated whole plant responses. This is accomplished through a complex systemic communication network involving a wide spectrum of physical, chemical and molecular components. The following section will explore the molecular bases of long distance osmotic signaling in plants from 3 types of signals, which are hydraulic, electric and chemical in nature. We will focus on inter organ signaling and more specifically on the root to shoot and shoot to root signals. Long distance communication has long been proposed to use the mass flow of sap within the vascular system. Accordingly, many molecules have been identified as traveling from root to shoots through the xylem vessels or from shoots to roots through phloem (reviewed in Ham and Lucas 2017; Winter and Kragler 2018).

### Molecular bases of long distance signaling

#### Hydraulic signal

**Fig. 3:**
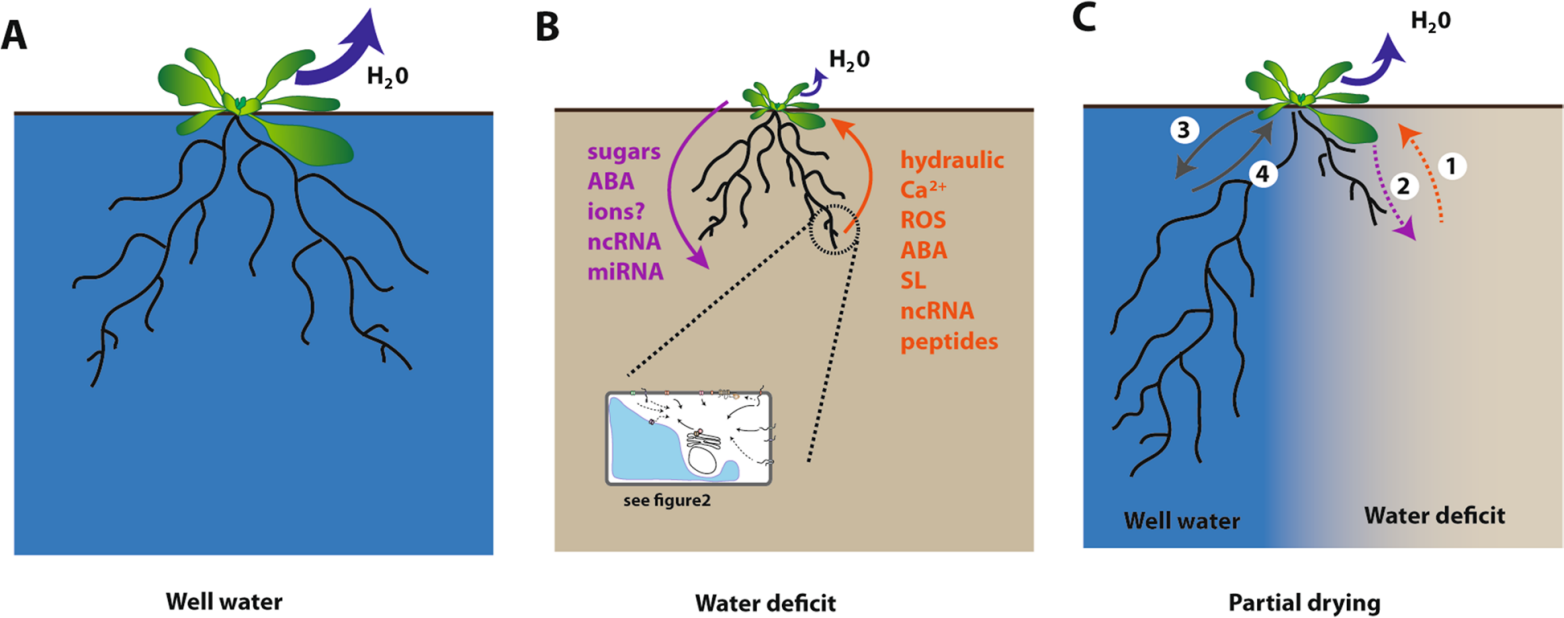
Long-distance and developmental response to homogeneous or local water deficit. **A** Soil water is absorbed by roots and moves through xylem vessels to the leaves where it is eliminated via transpiration (blue arrow). **B** When plants experience water shortage, they first dramatically reduce transpiration and modify root and shoot growth according to water availability. Roots, which perceive locally the osmotic stress as described in Fig. 2, activate long-distance signaling (orange arrow) conveyed by hydraulic signals or a wide range of molecules, including calcium (Ca^2+^), Reactive Oxygen Species (ROS), phytohormones (Abscisic Acid (ABA), Strigolactones (SL), etc), non-coding RNA (ncRNA) and peptides. In return, water deficit induces a shoot-to-root signaling (violet arrow) that relies on a set of molecules including sugars, ABA, ncRNA and micro RNA (miRNA). **C** When plants encounter a local water deficit also named partial root zone drying (PRD) in agronomy, the transpiration rate is reduced but not as severely as under a uniform water deficit. As a consequence, shoot development can be maintained or has a limited reduction depending on the intensity and duration of the local water deficit or on the plant developmental stage. Root growth in the drying part is strongly repressed whereas it is maintained or stimulated in the wet part through a compensatory growth stimulation. It was proposed that, during PRD, roots are sensing the local low water potential in the drying soil resulting in a reduction in cell turgor, then transmitting the signal to the shoot (arrow 1). In return, a shoot-to-root signal (arrow 2) represses root growth. Since root development and water uptake are stimulated in the well-watered part, the existence of both a shoot-to-root and a root-to-shoot signaling can be hypothesized (arrows 3 and 4). Besides ABA, the nature of other putative signals remains totally unknown

Water absorbed by roots is axially transported along the xylem towards shoots down the plant water potential gradient. Leaf water potential, which is the lowest in the plant, induces a tension in the vessels. Thus, any physical damage or disturbance that breaks the integrity of the water column present in the xylem vessels can release this tension, thereby inducing a pressure change that will almost instantly be transmitted through the vasculature. This physical signaling named hydraulic signaling has been proposed several decades ago to be involved in the fast response to leaf wounding (Houwink 1935) (Fig. 3B). The pressure wave generated through this process may be involved in the propagation of chemicals from the wounded tissue or directly trigger mechanical systemic signals in sensitive tissues (Farmer et al. 2014; Evans and Morris 2017). However, wounding experiments suggest that the hydraulic signal is moving up to 10,000 times faster than the observed propagation of the Ca^2+^ in tissues, thereby challenging the idea of a chemical propagation (Evans and Morris 2017). Nevertheless, more recent work suggests that change in hydraulic pressure induced by wounding is abolished in *rbohD* (NADPH oxidase) and *glr3.3 glr3.6* (glutamate receptor-like protein) (Fichman and Mittler 2021)*.* This suggests that hydraulic signal is not solely determine as a physical signal. In response to water deficit, the turgor pressure of leaf cells rapidly declines when roots experience water shortage. A drop in soil local water potential can also modify root turgor pressure and ultimately xylem tension. These changes can propagate from root to shoots as a so-called hydraulic signal (Christmann et al. 2007; Christmann et al. 2013). Alterations in root turgor pressure were also found to induce local accumulation and signaling of the well characterized water stress hormone Abscisic Acid (ABA) both in root and shoots (Christmann et al. 2007) indicating that hydraulic signaling may somehow be correlated to ABA signaling. However, a rapid response of stomata to water deficit was also observed in the ABA biosynthesis mutant (*aba2*) or signaling mutant (*abi1*) indicating an ABA independent signal (Christmann et al. 2013). In addition, grass stomata have subsidiary cells coupling osmotic and turgor adjustment to fasten stomatal movements (Franks and Farquhar 2007; Raissig et al. 2017). The action of hydraulic signals transmitted through the plant vasculature to the stomata thereby represents a rapid signaling mechanism well correlated to plant architecture and anatomy. However, whether the putative hydraulic signal is translated locally into chemical signals like ABA or acts locally through as yet unknown leaf structures remains an experimental challenge and a matter of vivid debate (Evans and Morris 2017; Farmer et al. 2020).

#### Electrical signal

Electrical signals have initially been identified in the folding of leaflets and petiole movements of *Mimosa pudica* in response to mechanical and other physical stimuli. These movements are driven by a turgor-powered motor and the rapid signaling involved appears linked to propagation of electrical signals that are moving through the sieve tube (recently reviewed by Johns et al. 2021). Plants maintain a -200 mV electrical potential difference across their plasma membrane through the action of ion channels, pumps (including H ^+^ -ATPases) and transporters. Transient depolarization and repolarization events with set amplitude, velocity and duration or variation of potential can convey key information about the strength and distance of the stimulus that triggered such electrical signals, within and between plant organs. These signals rely on fluxes of ions including K^+^, Cl^−^, H^+^ or Ca^2+^, the latter being the most documented. Electrical signaling has been reported to be involved in the response to heat, cold, touch, salt, flooding, hypoxia, osmotic stress, and drought (reviewed by Wilkins et al. 2016; Kudla et al. 2018). To explain such wide range of responses, it was hypothesized that the Ca^2+^-permeable channels controlling cell influx contain a stress-specific fingerprint that varies in amplitude, timing, and frequency and can be formed by the activation of different Ca^2+^ channels depending on the different stresses (Wang et al. 2019). In the case of drought, Ca^2+^ has been reported to move up in the transpiration stream and may couple Ca^2+^ supplied from the roots to stomatal response in the leaves (Han et al. 2003) (Fig. 3B). Storti et al. (2018) also measured Ca^2+^ waves propagation in response to osmotic stress in the non-vascular moss *Physcomitrella patens*. The lower propagation rate compared to the one measured in Arabidopsis confirms the cell-to-cell and vascular dependent routes. Such mobile Ca^2+^ has recently been found to act through a plastid based receptor system in the guard cells that itself may further integrate these responses with other stimuli as observed during photoacclimation (Cutolo et al. 2019).

ROS accumulation and calcium production are closely interconnected and enhance each other during abiotic stress. For instance, superoxide produced by RBOH D activates calcium channels, which activate the vacuolar calcium channel TWO PORE CHANNEL1 (TPC1). This releases Ca^2+^ into the cytosol which in turn enhances the activation of RBOH protein D (Evans et al. 2016). This calcium/ROS feedback loop is likely instrumental for propagation of the ROS and Ca^2+^ waves during salt stress and a proper acclimation response. However, until recently, it remained unknown how ROS are perceived by cells to trigger calcium waves. The Leucine-rich-repeated receptor kinase (HPCA1) was identified from a forward genetic screen for impaired ROS-induced Ca^2+^ waves (Wu et al. 2020). Via covalent modification of its extracellular cysteine residues, HPCA1 is autophosphorylated, activated calcium influx and is required for stomata closure after H_2_O_2_ treatment. In a preprint, HPCA1 appear to be also necessary for propagation of ROS/Ca waves in response to high light signal (Fichman and Mittler 2020). Drought and high temperatures both induce calcium and ROS waves across the plasma membrane, but no in-depth mechanistic analysis on long distance propagation has been performed yet. The many different origins of ROS and corresponding Ca^2+^ waves suggest sophisticated signaling mechanisms. Thus, integrative studies are still needed to sort between the common and specific ROS-calcium signaling pathways that contribute to the specificity of plant responses to different stress conditions.

#### Sugars and long distance signaling

Drought, high salinity or temperature dramatically modify the metabolic profiles of plants (Urano et al. 2010; Cramer et al. 2011). It has long been proposed that uncharged metabolites such as proline or glycine-betaine, accumulate to promote water retention in the plant tissues without interfering with normal metabolism (Verslues and Sharma 2010). They, in concert with other specialized/secondary metabolites, also act as free radical scavengers removing excess ROS and reestablishing a cellular redox balance (reviewed by Takahashi et al. 2020). Sucrose, glucose, and fructose also highly accumulate in roots upon exposure to drought suggesting they can replace other osmolytes as major compatible solutes (reviewed by Takahashi et al. 2020). More recently, sugars have emerged as key players during shoot to root communication in response to water stress (Fig. 3B). In Arabidopsis, phloem transport of sucrose is mainly achieved through the activity of sugar transporters. Phloem loading of sucrose from photosynthetic leaf mesophyll cells is mediated first by the vasculature-localized sugar transporters SWEET11 and 12. They move sucrose from parenchyma cells into the apoplast where it is loaded into phloem companion cells by SUC2 (Chen et al. 2012). In the root, sucrose is unloaded either through an apoplastic pathway (via SUC/SWEET sugar transporters) or through a symplastic pathway (via the hydrostatic pressure) (Milne et al. 2018). Very recently Chen et al. (2022) showed that both SWEET11 and 12 are phosphorylated by drought or ABA activated SNF1-RELATED PROTEIN KINASE 2 (SnRK2) protein kinases, which enhance sucrose export to roots. This increased allocation is modulated by ABA signaling, promotes root development and increases foraging. The putative link between this sucrose dependent root growth stimulation and the SnRK1–target of rapamycin (TOR) energy signaling pathway remains to be elucidated. Indeed, sucrose accumulation may release the TOR1 dependent repression of transcription factors such as bZIP1, bZIP11 and bZIP53 or local auxin accumulation in primary root tip thereby promoting root growth (Hartmann et al. 2015; Weiste et al. 2017). Under drought stress, ABA activated SnRK2 can however phosphorylate TOR, thereby modulating the tradeoff between plant growth and drought response stress (Wang et al. 2018). A similar role for TOR can also be considered for primordia initiation through the regulation of WOX7, a sucrose dependent repressor of the cell cycle gene CYCD6 (Kong et al. 2016).

ABA also induces trehalose accumulation which in turns inhibits root growth (Wang et al. 2020). Finally, trehalose 6-phosphate (Tre6P) has also been demonstrated as a key regulator of source-sink relationships (Figueroa and Lunn 2016). In Arabidopsis, Tre6P is produced in the phloem and stomatal guard cells (Fichtner and Lunn 2021). Tre6P synthase (TPS), the enzyme that synthesizes Tre6P, plays a key role in the nexus between sucrose and Tre6P, operating in the phloem loading zone of leaves. Tre6P has a dual function as a signal molecule and homeostatic regulator of sucrose levels in plants in response to abiotic stresses such as drought. Most flowering plants contain barely detectable amounts of trehalose compared to sucrose. Despite the low amount of Tre6P, overexpression of TPS and TPP enzymes led to severe growth and developmental defects suggesting that changes in the level of Tre6P, the intermediate in the pathway, rather than in the level of trehalose itself, were responsible for the phenotypes. The concept that Tre6P functions primarily as a signal and regulator of sucrose levels in plants supports the existence of systemic signals for source-sink coordination (Fichtner et al. 2020; Fichtner and Lunn 2021). In growing sink organs, Tre6P would regulate the utilization of sucrose for growth and the accumulation of storage reserves, in part via complex interactions with TOR (Figueroa and Lunn 2016).

Taken together, shoot-originated sucrose and its underlying signaling network involving ABA and other phytohormones, SnRK2, TOR and potentially Tre6P seems to be involved in the fine tuning of root development under drought. Yet, further exploration is needed to better characterize the complex interactions shaping these signaling networks.

#### Hormones

Several hormones have been identified both in xylem and phloem sap and have been proposed to play a key role in the coordination of systemic stress response (Fig. 3B). Among these, ABA has been identified for long as a master regulator of plant responses to drought-, salt-, osmotic-, and freezing-based water limitations. ABA mediates drought stress response and resistance by regulating stomatal closure and stress responsive gene expression (Cutler et al. 2010). The involvement of ABA in drought responses has been extensively documented (recently reviewed by Kuromori et al. 2022) and will not be detailed here. In response to drought stress, ABA accumulates in all plant tissues but its accumulation is dramatically enhanced in leaves and more specifically in the vasculature of leaves as most of the ABA biosynthesis genes are expressed in this tissue (Kuromori et al. 2018). In addition, several ABA transporters are predominantly expressed in vascular tissues. Thus, long distance transport of ABA can occur through both xylem (Davies and Zhang 1991; Schachtman and Goodger 2008; Jiang and Hartung 2008) and phloem (Zhong et al. 1996), with reports of both root and shoot derived pools during water stress (Hartung et al. 2002; Manzi et al. 2015). Although the vasculature and apoplastic areas are described as the typical routes for systemic signaling, it is not completely understood how ABA movement from tissue to tissue is regulated. Recent studies have suggested that signaling peptides could be involved in the ABA dependent long distance signaling (see below).

Strigolactones (SL) form the most recently discovered class of phytohormones. ABA and strigolactones share a carotenoid precursor, from which strigolactone synthesis proceeds through a partially known series of enzymes to produce bioactive strigolactones (reviewed by Waters et al. 2017). Strigolactones have been shown to modulate several aspects of root and shoot development and interactions with rhizosphere organisms (Al-Babili and Bouwmeester 2015; Lanfranco et al. 2018). They also are thought to be involved in nutritional and abiotic stresses (Mostofa et al. 2018). Several dicot plants with defective strigolactone synthesis or signaling are hypersensitive to drought, salt and osmotic stress while exogenous application of SL reinforce drought tolerance in many species (Cardinale et al. 2018). In Arabidopsis, *Lotus japonicus* and tomato, SL positively control stomatal movements. Indeed, mutants in SL biosynthesis exhibited reduced stomatal closure (Ha et al. 2014; Liu et al. 2015; Visentin et al. 2016) whereas enhanced closure and drought tolerance was observed in plants treated with exogenous SL or over producing SL (Lv et al. 2018; Visentin et al. 2016; Zhang et al. 2018). Using grafting experiments in tomato, Visentin et al. (2016) confirmed that drought results in reduced SL accumulation in roots and over accumulation in shoots. Their experiments demonstrated that under-accumulation of SL in roots is responsible for its over accumulation in shoots. Thus, SL was identified as a long distance signal for drought dependent stomatal closure. SL acts on stomatal closure both in an ABA dependent and an ABA independent manner. The ABA-dependent pathway relies, at least in part, on ABA synthesis, transport and/or sensitivity. Accordingly, SL depletion decreases sensitivity to exogenous ABA in several species (Lv et al. 2018; Visentin et al. 2016). On the other hand, treatment with the synthetic SL analogue GR24 increases sensitivity to ABA in tomatoes (Visentin et al. 2016). Recently, Visentin et al. (2020) identified SL as a molecular component linking drought to miR156 accumulation and integrated miR156 in a model that links SL and ABA in tomato.

Also, it should be noted that the above-described model may be dependent on drought stress intensity and may be restricted to dicot plants. In rice, for instance, most SL biosynthetic mutants produce more ABA than the wild-type and thus are more resistant to drought (Haider et al. 2018). Finally, the local and systemic effects of drought-dependent under accumulation of SL in roots remain poorly documented.

Besides ABA and SL, several other hormones that have been identified in xylem and phloem sap (reviewed by Koenig and Hoffmann-Benning 2020) have been proposed to participate in plant adaptive responses to water deficit. For example, Brassinosteroids and Auxin are involved in the regulation of root and shoot growth under drought (reviewed by Gupta et al. 2020). Cytokinins were reported to mitigate water deficit growth limitation and stabilize yield (Hai et al. 2020). Finally, methyl jasmonate, that is well known for mediating long distance signaling in response to wounding or biotic stresses induces stomatal closure similar to ABA (Huang et al. 2017b). Jasmonoyl-isoleucine (JA-Ile) was also associated with osmotic signals, since it accumulates in cells under hypo-osmotic condition and conversely is reduced under hyperosmotic stress (Mielke et al. 2021, Science advances). The involvement of multiple hormonal pathways in different plant tissues emphasizes the complexity of plant hormonal responses to drought (reviewed by Hai et al. (2020); Sirko et al. (2021) and Kuromori et al. (2022)) and their role in long distance signaling of water deficit most often remains to be established.

#### Nucleic acids

Nucleic acids, more specifically RNA species, are among the most well studied long distance signaling molecules. In their extensive recent review Gelaw and Sanan-Mishra (2021) collected a large panel of non coding RNA that are differentially expressed in response to drought suggesting a central role in signaling (Fig. 3B). Representatives of all types of RNA, such as mRNA, micro RNA (miRNA), small interfering RNA (siRNA), and other non-coding RNA (ncRNA) have been identified in the sap of many plant species including Arabidopsis, rice, barley, maize, pumpkin and many others (Kehr and Kragler 2018; Liu and Chen 2018; Guo et al. 2013; Westwood 2015). As systemic signals, mobile RNAs are regulatory elements by which plants respond to dynamic changes in the environment. Breakthroughs studies on the regulatory mechanisms of long-distance RNA transport have been made in recent years. For detailed reviews of RNA trafficking see the publications of Ham and Lucas (2017); Kehr and Kragler (2018); Liu and Chen (2018) or Zhang et al. (2021a, b). While the existence of mRNAs and ncRNAs in the vascular system hints at their mobility and possible role, identification alone does not conclusively prove movement or physiological function. By using homo (intra species) and hetero (inter species) grafting, many studies in model plants, crops and woody plants have provided evidence for mobile mRNAs (Notaguchi et al. 2015, Thieme et al. 2015, Liu et al. (2020) and lnRNA (Zhang et al. 2009; Zhang et al. 2021a, b). Similar results were also reported for miRNAs (Pagliarani et al. 2017; Tolstyko et al. 2019). Data suggest that environmental conditions, including water deficit, may affect transcript mobility, independent of changes in gene expression (Thieme et al. 2015; Zhang et al. 2016; Tolstyko et al. 2019). In line with these results, Zhang et al. (2016) demonstrated that the transmissibility of mobile mRNAs is related to tRNA-like structural elements (TLs). TLs can modulate mRNA transport and are necessary for mediation of mRNA movement across the grafting junction. Recent studies by Yang et al. (2019) have shown that RNAs can contain 5-methylcytosine (M5C) and that this methylation can regulate long distance mRNA transport. Other studies have identified additional selective mechanisms including specific sequence motifs. For instance, untranslated regions or cis acting elements at 5′ end appear to influence transcript stability for transport, impact delivery to distal tissues and translation level (Banerjee et al. 2009; Li et al. 2009). Using a long stem hetero grafting system, Xia et al. (2018) showed that the abundance and the structure of mRNAs were degraded during the trafficking, suggesting putative modulation to stress response. Furthermore, using an elegant triple hetero grafting approach with a potato and a *Nicotiana benthamiana* scions grafted onto a tomato root stock, it was shown that mRNA transferred from the scion to the stock can be transported back to the scion after being transported again to shoots in a “shoot-root-shoot” cycling process (Xia et al. 2018; Wang et al. 2021). Taken together, the wide range of molecules, transport regulation and processing steps point to an extremely complex regulation network of long distance signaling.

#### Proteins and peptides

Several proteomic studies have identified up to thousands of proteins and peptides both in the xylem and phloem sap (Rodriguez-Medina et al. 2011; Carella et al. 2016). Recent analyses have elucidated the molecular mechanisms involving the long distance regulatory protein Flowering Locus T (FT). This 20 kDa protein is phloem mobile from leaves to shoot apical meristem where it acts as a florigen signal inducing flowering transition (Putterill and Varkonyi-Gasic 2016). Besides this well documented example very little is known about protein signaling functions. Nevertheless, mobile peptides form an important class of molecules possibly involved in long distance signaling in response to abiotic stress. Over 7000 small open reading frames (ORF) can be expressed in response to a wide range of environments in Arabidopsis suggesting a multitudes of functions (Hanada et al. 2007; Hanada et al. 2013; Ren et al. 2021) (Fig. 3B). A recent study has shown that the CLAVATA3/EMBRYO-SURROUNDING REGION- related25 (CLE25) peptide is a mobile, long-distance signaling molecule originating from roots and sensed in leaves where its perception induces ABA biosynthesis and stomatal closure (Takahashi et al. 2018). CLE25 was found to be produced in root vasculature and moves from root to shoots through the vascular system. In leaves, it is recognized by the BARELY ANY MERISTEM1 (BAM1) and BAM3 receptors that induce NINE-CIS-EPOXYCAROTENOID DIOXY- GENASE3 (NCED3) expression to enhance ABA accumulation in leaves. This, in turn, regulates stomatal aperture in response to dehydration stress thereby reducing water losses (Takahashi et al. 2018). Although the mechanisms involved in regulating CLE25 production, loading and unloading, and CLE25–BAM ABA production remain to be elucidated, these results identify the CLE25–BAM long-distance signaling system as a key component of drought stress response. Interestingly, other CLE peptides (CLE 9/10) were found to be involved more locally in stomatal development and regulation (Qian et al. 2018). CLE9 has also a role in stomatal closure in response to drought in a ABA dependent manner (Zhang et al. 2018). While this peptide-dependent mechanism is definitely slower than hydraulic signaling, it may be an important component during mid-term and long-term dehydration stress responses. It may also be involved in the response to gradual soil drying as generally observed under natural conditions. One can also expect that the CLE-BAM long distance signaling mechanism plays a crucial role under heterogeneous water supply to the roots, when part of the root system experiences water deficit while other parts are well-watered. Interestingly, a similar mechanism has also been reported in response to nitrogen deficiency. Local root nitrate deficiency induces production of C-TERMINALLY ENCODED peptides (CEP) that are translocated to shoots where they are recognized by the CEP Receptor (CEPR). This in turn induces the local accumulation and transport to roots of the CEP DOWNSTREAM1 and 2 (CEPD1 and 2) polypeptides (Ohkubo et al. 2017). Thus, the CEP–CEPR–CEPD system mediates long-distance signaling from roots to shoots to roots in order to transmit the nitrogen deficit signal throughout the whole plant. Interestingly, a similar roots-to-shoots-to-roots signaling has also been documented during the control of nodule formation in the legume plant *Lotus Japonicus*. This systemic regulation that involves cytokinins connects nutritional status, development and hormonal signalization (reviewed by Okamoto et al. 2016; Ferguson et al. 2019). Similar mechanisms have not yet been reported in response to drought but several works identified CEPs which expression is induced by water deficit (Smith et al. 2020) suggesting they could also exist to adjust and coordinate development and water homeostasis.

### Physiological and developmental response to soil water heterogeneity

Heterogeneity of water availability around plant roots is a widespread phenomenon, occurring both in natural and agricultural environments (Fig. 3C). At macro-scale, when rainfall or irrigation are limited, the upper soil layers typically dry faster as they are exposed to evaporation and water uptake by plant roots where their density is the highest. This results in an uneven vertical distribution of soil moisture with depth (Beff et al. 2013; Mohanty 2013; Zhang and Davies 1989). Water availability can also be horizontally heterogeneous, because of plant competition and local soil water retention (Ivanov et al. 2010). Due to the high heterogeneity in structure and composition of soil, water can also be heterogeneously distributed at the local or micro scale. One of the most striking examples is the presence of macropores that locally induce an extreme water deficit (see extensive reviews by Beven and Germann 1982 and Jarvis 2007). Recent studies have identified the molecular mechanisms that allow roots to locally sense moisture gradients and direct their growth or formation of new lateral roots towards increased water availability. These local adaptations to water availability are referred to as hydrotropic response (Dietrich et al. 2017) and hydro-patterning (Bao et al. 2014; Orosa-Puente et al. 2018), respectively. Similarly, Orman-Ligeza et al. (2018) described a process called xerobranching which accounts for the repression of lateral root formation when a root grows through a large air-filled soil macropore. In all cases, root tip growth and root branching are positioned towards regions of higher water availability. While many mechanistic details are being uncovered from laboratory experiments, it will be fascinating and critical to understand how these growth and development adjustments mutually interact and operate during growth of roots in real, drying soils and how they allow optimizing soil foraging and water uptake at the whole plant level.

As an illustration, irrigation strategies have been introduced in agriculture to deliberately create a heterogeneous distribution of soil water, both spatially and temporally. One of these techniques called partial root zone drying (PRD), aims at locally irrigating a limited part of the root system while the other part of the root system faces water deficit (Dbara et al. 2016; Fu et al. 2017; Puértolas et al. 2015; Stoll et al. 2000). In agricultural production, PRD usually requires a regular permutation of watered and unwatered sides, to ensure the survival of the entire root system, thus forming a cycle of root drying and wetting. This technique was found to improve water use efficiency (WUE) compared to irrigation at field capacity in many greenhouse and field trials (Iqbal et al. 2020). For several crops, such as cotton (Du et al. 2008; Tang et al. 2005), tomato (Kirda et al. 2004; Zegbe et al. 2006), hot pepper (Kang et al. 2001; Shao et al. 2008), grape (De la Hera et al. 2007), and pear (Kang et al. 2002), PRD improved WUE without significant effect on yield compared to fully irrigated plants. However, studies in other crops showed more contrasted results. In maize for instance, some studies showed improved WUE with no reduction or even increase of grain yield (Fu et al. 2017; Kang et al. 2000; Sepaskhah and Khajehabdollahi 2005; Sepaskhah and Parand 2006) whereas another showed a lowered grain yield (Hakeem et al. 2016).

It is proposed that, during PRD, the drying roots sense the locally low water potential resulting in a reduction in cell turgor and thereby inducing a systemic partial stomatal closure and reduced leaf expansion. Concomitantly, roots in wet parts of the soil absorb large amounts of soil water to maintain elevated water content in shoots, consequently increasing water use efficiency (Kang and Zhang 2004; Christmann et al. 2013). Alteration of leaf growth and development and changes in metabolism can hinder the use of carbon, energy, and allocation of the plant’s photoassimilates, which are then preferentially reallocated to roots enhancing root expansion in the irrigated part (Taiz and Zeiger 2006). Yet, the nature of the local and systemic signals involved in responses of the whole root system remains unknown. At the functional and molecular level, temporal PRD study in a riparian *Melaleuca* species showed the root hydraulic conductance and aquaporin abundance to be rapidly increased in the wet side (within 24 hours) after local dehydration of the root system (McLean et al. 2011), suggesting a systemic compensation and regulation of water uptake during PRD.

Several investigations have been carried out using a split-root system to mimic PRD and explore the local and systemic signaling governing PRD. ABA is the most studied non-hydraulic signal that may regulate and coordinate the underlying developmental and functional responses. Early studies have shown that tomatoes under root-zone water deficit had a lower stomatal conductance and greater root hydraulic conductivity due to overproduction of ABA (Thompson et al. 2007). However, Liu et al. (2008) noted that PRD plants had a lower stomatal conductance and similar photosynthesis compared to fully irrigated plants. Yet, the xylem sap ABA concentration of PRD plants was not higher than fully irrigated plants in the first day of PRD, making ABA questionable as the root to shoot signal in the early stage of PRD.

The different effects of water heterogeneity within the root zone have also been explored in pot experiments (Puértolas et al. 2013; Puértolas et al. 2015). In a set of experiments using beans (Puértolas et al. 2013) or potatoes (Puértolas et al. 2015) grown in soil columns that received different irrigation treatments to induce distinct vertical soil moisture gradients, it was observed that root ABA concentration and root water potential were homogeneous within the different root parts. On the contrary, horizontal heterogeneous soil moisture induced much higher ABA accumulation in the roots (Puértolas et al. 2015). These results and others on barley (Martin-Vertedor and Dodd 2011) challenge a direct correlation between local water deficit and ABA signaling. Many molecules including sugar, proline and other metabolites (Abdallah et al. 2019; Iqbal et al. 2019; Raza et al. 2017) or proteins (Sadak et al. 2019; Sadak et al. 2020) were found to be produced and over accumulate under PRD irrigation conditions (Fig. 3C). However, there is no direct evidence yet to prove a possible role in systemic signaling. Furthermore, the role during PRD of other putative signals such as small RNA, microRNA, ncRNA and peptides has not been explored yet (Fig. 3C).

## Conclusion and prospects

In recent years, much progress has been made in deciphering the mechanisms for sensing and signaling water deficit. The identification of several sensing molecules exemplify the central role of osmosensing in plant. But, how these molecules interconnect remains mostly unexplored. They might act in parallel pathway reflecting the diversity of osmotic signal that cells have to face. Alternatively, they could share some redundancy in the perception machinery or in triggering the downstream signaling. Unfortunately, our current knowledge remains too fragmented to assess the proper kinetic of events. Interestingly, most of the molecular actors described so far are localized to the plasma membrane, as it is suspected to be the ideal place for water sensing. Nevertheless, we realize that changes in cell volume or turgor could be perceived in subcellular compartments or structures deprived of membranes. For instance, liquid-liquid phase separation (LLPS) is a process where two liquids can be separated into non-miscible phases depending on concentration and which can be modulated by physico chemical alterations of the system (Cuevas-Velazquez and Dinneny 2018; Korkmazhan et al. 2021). LLPS can happen at many cell locations including contacts with a membrane but also in the cytoplasm or nucleoplasm, where it was originally discovered (Nucleolus, Cajal bodies, nuclear speckles). In animal cells, processing bodies (PBs) containing mRNA-decapping enzyme 1A (DCP1A) are examples of subcellular compartments that are deprived of membranes and rapidly phase separate under hyperosmotic stress while dissolving back upon isotonic rescue (Jalihal et al. 2020). These PBs sequestrate pre-mRNA cleavage factors from actively transcribing genome loci (Jalihal et al. 2020). This example provides a mechanical framework for gene regulation under hyperosmotic stimulation. Similarly, the apoptosis signal-regulating kinase 3 (ASK3) is inhibited by phase separation under hyperosmotic stress (Watanabe et al. 2021). In plants, phase separation can be associated to many processes like regulation of flowering time, temperature sensing, and auxin or SA signaling, (Fang et al. 2019; Powers et al. 2019; Zavaliev et al. 2020; Jung et al. 2020). Regarding water sensing, FLOE1, a prion like structured protein, undergoes phase separation in vitro and during seed imbibition (Dorone et al. 2021). The biophysical state of FLOE1 modulates its biological activity in suppressing seed germination under unfavorable environments. Moreover, it was found that natural variation in the coding sequence of FLOE1 is associated with adaptive germination strategies in natural populations (Dorone et al. 2021). These findings on significance of LLPS in biology open new avenues to re-investigate the molecular mechanisms of plant osmotic sensing.

Like for osmotic perception, a large and ever increasing number of molecules have been proposed to act in local and long distance signaling of water availability. Strikingly enough, most of these signaling molecules and their corresponding receptors are likely to possess more functions than those that were originally assigned, revealing a complex array of interactions and interplays. Moreover, these molecular mechanisms have been identified in a limited number of model plants cultivated under highly controlled and often artificial growth conditions. In contrast, responses at the whole plant level have been mostly investigated in crops or understudied species. This is particularly striking for PRD where researches were conducted in crops/trees grown in a wide range of stresses (localization intensity, duration). Thus, it is difficult to integrate all available information to build a systemic signal network for plant response to heterogeneous water distribution. Furthermore, many of the signal molecules and mechanisms identified under homogeneous water deficit condition have not been or very partially investigated under heterogeneous water distribution.

Accordingly, little is known about the nature, the temporality and the function of sensing and local and systemic responses to heterogeneous water availability. We believe that studying rapid and long term responses to local water deficit in model species and under controlled conditions should lead to breakthroughs in the identification of the molecules and of their interactions that trigger plant acclimation responses. Identifying the main actors that trigger, prime or coordinate plant responses to water availability will provide a first step toward improving the efficiency and coordination of these responses. In the long term, these studies will allow identifying novel breeding targets to enhance crop tolerance to drought and develop new varieties that are well adapted to water saving irrigation strategies.

## Data Availability

Not Applicable.

## Abbreviations

ABA: Abscisic Acid
ABI: Abscisic Acid Insensitive
APX: Ascorbate Peroxidases
ASK3: Apoptosis Signal-regulating Kinase 3
At: *Arabidopsis thaliana*
BAM1: Barely Any Meristem 1
bZIP: Basic region, leucine ZIpper
Ca^2+^: Calcium
CAT: Catalases
CEP: C-terminally Encoded Peptides
CEPD: CEP Downstream
CHO: Chinese Hamster Ovary
Cl: Chloride
CLE25: CLAVATA3/EMBRYO-SURROUNDING REGION- related25
CrRLK: *Catharanthus roseus* RLK1-Like kinases
CSC1: Calcium-permeable Stress-gated cation Channel 1
CYCD: Cyclin D
DCP1A: mRNA-Decapping enzyme 1A
EF domains: Helix–loop–helix structural domain
EMS: Ethyl Methane Sulfonate
ECA1: Endoplasmic reticulum Ca^2+^ pump
E PCSs: ER-plasma membrane Contact Sites
FER: FERONIA
FLOE: “Sheet of floating ice”
FT: Flowering Locus T
GLR: Glutamate Receptor-like protein
GPX: Glutathione Peroxidases
H ^+^: Hydrogen
H_2_O_2_: Hydrogen peroxide
HEK: Human Embryonic Kidney
HK: Histidine Kinases
HOG1: High Osmolarity Gycerol 1
HPCA1: Hydrogen Peroxide sensor
K^+^: Potassium
KEAs: K^+^ Exchange Antiporters
LLPS: Liquid-Liquid Phase Separation
lnRNA: Long non coding RNA
LRK: Lectin Receptor-Kinase
LRR: Leucin Reach Repeat
M5C: 5-methylcytosine
MAP kinase: Mitogen-Activated Protein Kinases
MCA1: Mid1-Complementing Activity
MIK2: MALE DISCOVERER 1-INTERACTING RECEPTOR-LIKE KINASE 2
MIZ1: MIZU-KUSSEI1
mRNA: Messenger RNA
miRNA: Micro RNA
MscS: Mesenchymal stem cells
MSL: MscS-like
NADPH: Nicotinamide Adenine Dinucleotide Phosphate Hydrogen
NCED3: NINE-CIS-EPOXYCAROTENOID DIOXY- GENASE3
ncRNA: Non-coding RNA
ORF: Open Reading Frames
OSCA1: REDUCED HYPEROSMOLALITY-INDUCED [CA^2+^] INCREASE 1
PBs: Processing Bodies
PCD: Programmed Cell Death
PRD: Partial Root zone Drying
PERKs: Proline-rich Extensin-like Receptor Kinases
PIP: Plasma membrane Intrinsic Protein
PM: Plasma Membrane
PRX: Peroxiredoxins
PTI: Pattern-Triggered Immunity
RALF34: Rapid Alkalinization Factor
RBOHD and F: Respiratory Burst Oxidase Homolog D and F family
Rho: Ras-homologous family proteins
RLKs: Receptor Like Kinases
RNA: Ribonucleic acid
ROP6: Rho of Plant 6
ROS: Reactive Oxygen Species
RSA: Root System Architecture
SA: Salicylic Acid
SCOOP12: SERINE RICH ENDOGENOUS PEPTIDE12
siRNA: Small interfering RNA
SL: Strigolactone
SLN1: Synthetic Lethal of N-end rule 1
SnRK: SNF1-RELATED PROTEIN KINASE
SOD: Superoxide Dismutases
THE: THESEUS
TIP: Tonoplast Intrinsic Protein
TLs: tRNA-Like structural elements
TOR: Target Of Rapamycin
TPC1: Two Pore Channel 1
TPS: Tre6P Synthase
Tre6P: Trehalose 6 Phosphate
TM: Trans Membrane
WAK: Wall Associated Kinases
WOX: Wuschel-like homeobOX
WUE: Water Use Efficiency
